# Career Experiences of Special Education Teachers in China: Implications for Teacher Well-Being and Workforce Support

**DOI:** 10.3390/bs16101844

**Published:** 2026-10-08

**Authors:** Meili Cheng, Jinxuan Wang, Yuqing Deng, Lu Chen, Yonggang Su, Xiuxiu Shen, Shuai Zhang

**Affiliations:** 1 School of Foreign Languages and Literature, Shandong University, Jinan 250100, China; 2 School of English and International Studies, Beijing Foreign Studies University, Beijing 100089, China; 3 Health Studies Research, Methodologies, Florence Nightingale Faculty of Nursing, Midwifery & Palliative Care, King’s College London, London SE1 8WA, UK; 4 School of Translation Studies, Shandong University, Weihai 264209, China; 5 School of Foreign Languages, University of Jinan, Jinan 250022, China

**Keywords:** special education teachers, teacher well-being, career experiences, professional meaning, occupational strain, institutional support, qualitative content analysis

## Abstract

Special education teachers’ career experiences span entry pathways, role enactment, professional meaning and strain, and institutional support, yet these dimensions are often studied separately. This qualitative study examined how 19 full-time special education teachers understood and connected these aspects of their careers. Participants included 17 women and 2 men with 2–33 years of teaching experience, each employed at a different public special education school. The schools were located predominantly in northern and eastern China, with fewer in coastal southern cities. Semi-structured interviews were analyzed using qualitative content analysis with inductive open coding and iterative abstraction. Three themes were generated: making special education a viable career; enacting professional capacity through role; and navigating meaning, demands, and support in special education work. Participants retrospectively linked career entry to the alignment of value-based commitment with viable employment pathways; they described professional capacity as enacted through specialist, pedagogical, rehabilitation-oriented, and relational work; and they discussed sources of professional meaning, occupational demands, and support needs in relation to development opportunities, recognition, and institutional support. Across participants’ accounts, personal commitments, professional role enactment, relational work, and institutional conditions were intertwined. These findings suggest areas for professional learning and workforce support and raise questions for future research on teacher well-being.

## 1. Introduction

Special education teachers (SETs) are central to educational support for students with disabilities (Brownell et al., 2010; Lee et al., 2018), yet sustaining this workforce requires more than filling vacancies. It also depends on how teachers enter the field, develop and enact expertise, understand their roles, and experience the rewards and demands of the work, and access conditions that support continued learning and retention (Billingsley, 2004; Billingsley & Bettini, 2019; Brownell et al., 2010). These issues are salient in China: in 2025, 2464 special education schools served 349,900 students, within approximately 927,000 students receiving special education across provision types, supported by 85,100 full-time SETs nationwide (Ministry of Education of the People’s Republic of China, 2026). National policy provides a framework for teacher preparation and recruitment: the 2015 professional standards define expected competencies; the 2021–2025 action plan supports university-based preparation and continuing training; and 2025 public recruitment guidance specifies vacancy announcements, eligibility screening, competence assessments, and certification requirements (General Office of the State Council, 2022; General Office of the Ministry of Human Resources and Social Security & General Office of the Ministry of Education, 2025; Ministry of Education of the People’s Republic of China, 2015). The action plan also recognizes uneven regional development, calling for provision tailored to local conditions and prioritizing funding for central and western regions (General Office of the State Council, 2022). Thus, national policies establish expectations for preparation and recruitment, while teachers’ access to these opportunities and supports may vary across local contexts.

Research on entry into special education shows that career choice reflects both value-based and contextual considerations, including social-utility values, intrinsic interest, self-perceptions, opportunities for professional growth, and family and cultural influences (Almughyiri, 2025; Lindqvist et al., 2021; Valdenegro-Fuentes & González-Sanzana, 2024). Therefore, this research suggests that entry cannot be reduced to intrinsic or extrinsic motives alone; it also depends on how personal commitments align with available preparation and employment pathways. Related adolescent research, although not specific to special education entry, adds context for the role of family and school influences. Among Italian adolescents, parental interference in career choices was associated with lower career decision self-efficacy and school engagement, as well as higher school anxiety, burnout, and depressive symptoms (Angelini et al., 2026). A study of Chinese adolescents linked parenting styles and parental involvement to academic engagement and burnout, with perceived parental support mediating some of these associations (Zhu et al., 2023). More broadly, a systematic review linked academic pressure with adolescent mental-health problems, most commonly depressive symptoms or a combination of anxiety and depressive symptoms (Steare et al., 2023). Research on special education preparation then turns to the knowledge and practices needed to enact the professional role effectively, including professional knowledge, instructional decision-making, implementation skills, and responsiveness to the contexts in which teachers work (Brownell et al., 2010; Oliver & Reschly, 2010; Theobald et al., 2022). Empirical work has examined classroom-management preparation and alignment between teacher preparation and literacy practice, while more recent research has broadened attention to collaboration and interaction, digital competence, and emotional competence (Calandri et al., 2025; Montenegro-Rueda & Fernández-Batanero, 2024; Oliver & Reschly, 2010; Takala et al., 2023; Theobald et al., 2022). Together, these studies portray professional capacity as a developing combination of specialist, pedagogical, relational, and adaptive resources (Brownell et al., 2010; Takala et al., 2023; Theobald et al., 2022).

What teachers can do is closely tied to how they understand and enact the special educator role (Ellis-Robinson et al., 2025; Kluge-Brück et al., 2026; Lin, 2026). This role varies by context: in inclusive schools, responsibility may remain specialized or become shared with general educators, while other settings emphasize support for students with severe and multiple disabilities or facilitation of peer relationships (Kluge-Brück et al., 2026; Lee et al., 2018; Sigstad, 2018). Professional identity is likewise shaped through personal values, role expectations, school contexts, and experience. Studies in China and the United States show that special educators negotiate identities and agency as service conditions and understandings of the role change (Ellis-Robinson et al., 2025; Lin, 2026). Moreover, the relational character of special education work further complicates the boundary between professional capacity and role (Aspelin et al., 2021; Stark et al., 2026). Experienced special educators have described positive teacher–student relationships as fundamental to their work, with relational competence realized through accepting, personal, and trusting relationships with students (Aspelin et al., 2021). Relational work also extends to collaboration with adults (Stark et al., 2026). Stark et al. (2026), for example, found that SETs used emotional labor strategies as a professional tool in interpersonal interactions and collaborative efforts with other adults. These studies position relational and emotional work within the performance of special educators’ professional roles rather than at the margins of practice.

Research on retention, burnout, and occupational well-being identifies preparation, school characteristics, working conditions, and teacher-level factors as recurrent influences on special education careers (Billingsley & Bettini, 2019; Brunsting et al., 2026). Working conditions, including administrative and collegial support, resources, compensation, job design, autonomy, and opportunities to teach effectively, have been linked to effectiveness, job attitudes, and intentions to stay or leave (Billingsley et al., 2020; Cancio et al., 2013; Conley & You, 2017). Burnout research emphasizes emotional exhaustion, depersonalization, and reduced personal accomplishment, while qualitative work shows how being overworked and underappreciated can connect everyday stressors with thoughts of leaving (Brunsting et al., 2014; Hester et al., 2020; Park & Shin, 2020). Moreover, research on SET well-being also addresses positive work evaluations and supportive resources rather than strain alone: studies of special educators have examined job satisfaction in relation to autonomy and alongside dimensions of burnout (Elsayed et al., 2025; Olsen & Mason, 2023), while evidence from China shows that social support is associated with special educators’ well-being and that emotional labor mediates this relationship (Wu et al., 2020). This literature supports examining positive and strain-related dimensions within the same analytic frame rather than treating well-being solely as the absence of strain (Channing & Wilson, 2026; Elsayed et al., 2025; Olsen & Mason, 2023; Wu et al., 2020). A qualitative study of U.S. educational leadership educators during the COVID-19 pandemic likewise documented blurred instructional–counseling roles, invisible care work, competing care obligations, and role strain, illustrating how caring work can have implications for educator well-being (Channing & Wilson, 2026). In China, extra-administrative workload has been associated with greater emotional exhaustion among primary and secondary teachers (Shen et al., 2025).

Professional learning provides one point of connection between professional capacity and broader career conditions (Pi et al., 2024; Woulfin & Jones, 2021). Woulfin and Jones (2021) found that special educators valued specialized learning opportunities but had limited access to ongoing, collaborative, and contextually relevant professional development; they argued that professional learning also contributes to occupational socialization and professional identity. Research on Chinese SETs likewise shows statistical relationships among professional identity, self-efficacy, perceived social support, and professional development (Pi et al., 2024). Other work shows how special education practice is being reconfigured in digitalized school environments (Holmgren, 2023). These studies suggest that professional development is not only a matter of training volume: access, contextual relevance, and opportunities to apply learning also matter (Pi et al., 2024; Woulfin & Jones, 2021).

Existing research has identified factors associated with special education teachers’ career choices, professional roles, working conditions, and professional learning (Billingsley & Bettini, 2019; Brownell et al., 2010; Lin, 2026; Lindqvist et al., 2021; Woulfin & Jones, 2021). In China, studies have addressed social support and well-being, professional identity and development, preservice identity construction, and novice teachers’ professional development (Feng & Kim, 2025; Lin, 2026; Pi et al., 2024; Wu et al., 2020). These studies provide evidence about particular aspects of teachers’ careers and relationships among selected constructs. However, less is understood about how practicing SETs in China connect their reasons for entering the profession with their accounts of everyday responsibilities, professional accomplishment, occupational difficulties, and support needs. In particular, how teachers describe the compatibility or tensions between what they value in special education and the conditions under which they carry out their work remains insufficiently understood.

Teachers’ accounts of valued work under particular working conditions are also relevant to teacher well-being. In this study, well-being serves as an interpretive lens rather than a measured outcome. Teacher well-being is multidimensional, encompassing physical and mental, cognitive, subjective, and social dimensions (Viac & Fraser, 2020). Within this framework, professional meaning captures teachers’ appraisal of their work’s significance and value (Steger et al., 2012), while strain captures the burden of occupational demands. Relational work comprises the interpersonal and emotional activities through which teachers enact their roles; it may be a source of connection and meaning as well as emotional demand (Hascher & Waber, 2021; Yin et al., 2019). Institutional support refers to resources that may shape how teachers manage demands and sustain professional relationships and valued work (Li et al., 2025). Thus, meaning and strain capture different qualities of teachers’ work experiences; relational work may involve both, while institutional support may shape how teachers experience and sustain their work. Using semi-structured interviews and qualitative content analysis, this study explored the career accounts of practicing SETs from different public special education schools in China. Two research questions guided the inquiry:

RQ1. How do practicing SETs in China describe their entry into the profession, professional capacity and roles, sources of meaning and strain, and future professional expectations and support needs?

RQ2. How do teachers connect these aspects of their careers, particularly when describing alignment or tensions between their professional commitments and the conditions of their everyday work?

## 2. Materials and Methods

### 2.1. Research Context and Participant Recruitment

Nineteen full-time SETs, each employed at a different public special education school, participated in the study; no two participants were employed at the same school. All schools were public institutions, and no participants worked in inclusive education settings within mainstream schools. Most schools were located in northern and eastern China, with fewer in coastal southern cities. To protect institutional anonymity, we do not report school names or more precise location details. We recruited participants through convenience sampling supplemented by snowball recruitment. We first contacted approximately 40 teachers through Xiaohongshu; five responded, and two agreed to participate. The remaining participants were recruited through professional or personal contacts: six were colleagues of the research team, two were friends, and nine were referred through acquaintances. Approximately five more teachers were proactively contacted through snowball recruitment, mainly following a recommendation from a school director in Northeast China. No participant withdrew after enrollment. Teachers who expressed interest were screened against the eligibility criteria before enrollment. We invited initial participants who were accessible through the research team’s professional networks and met the eligibility criteria. After each interview, we invited participants to share study information with potentially eligible colleagues. Interested teachers contacted the research team directly and were screened against the eligibility criteria before enrollment.

In this study, SETs were defined operationally as full-time teachers working in special education schools if they (a) were employed full-time as SETs; (b) had at least one year of teaching experience in a special education context; and (c) were directly involved in the instruction, rehabilitation, or educational support of students with disabilities. However, participants were not required to have the same academic major or teaching responsibilities. We excluded five respondents because they did not meet these criteria; they included school administrators; preservice or intern teachers; part-time personnel; and staff without direct responsibilities for instruction, rehabilitation, or educational support. Participants served as subject teachers, life-skills teachers, or rehabilitation teachers. Teaching experience ranged from 2 to 33 years. All participants had worked continuously in special education throughout their teaching careers; thus, their reported teaching experience indicates their approximate duration in the field and, in relation to the 2025 interview period, their approximate period of entry into special education. Age was not collected, and participants were not formally classified by career stage. Thirteen participants held bachelor’s degrees, five held master’s degrees, and one held a secondary normal school diploma. Their teaching responsibilities included Chinese language, mathematics, music, physical education, life-skills education, health education, arts and crafts, and rehabilitation. Most had received professional preparation in special education; others had backgrounds in education, rehabilitation, early childhood education, literature, or the arts. This variation provided perspectives from teachers with different forms of educational preparation, career experience, and professional responsibility. Table 1 summarizes participant characteristics.

### 2.2. Data Collection

We conducted individual semi-structured interviews in Chinese between July and September 2025. Questions were open-ended and were followed by probes asking participants to clarify meanings, describe concrete experiences, and explain why particular events or conditions mattered to them. The broad areas structured elicitation rather than prescribing the analytic outcomes. After the first two interviews, we made minor adjustments to the interview guide, primarily clarifying the wording of several questions, changing their order to improve the flow of the interview, and refining follow-up prompts to elicit more specific descriptions of participants’ experiences. The core questions and substantive interview areas remained unchanged; therefore, the first two interviews were retained in the analysis. Interviews were all conducted through Tencent Meeting, depending on participants’ preferences and availability. Table 2 presents the semi-structured interview guide.

The first author and the third author conducted all interviews. Both were PhD students in applied linguistics with research experience in special education and had training in qualitative interviewing and experience in education research. The interviewers held no supervisory or evaluative role in relation to participants; any prior contact was limited to professional-network connections used for recruitment. With written informed consent, we audio-recorded all 19 interviews. Interviews lasted 60–80 min, with an average duration of approximately 70 min.

Recruitment, interviewing, and preliminary analysis proceeded concurrently. After each interview, the research team reviewed the transcript and analytic notes to assess whether later interviews were extending, complicating, or repeating the developing analytic patterns relevant to the research question. By the completion of Interview 17, the most recent interviews were largely repeating rather than materially extending these developing patterns. We conducted two additional interviews to examine whether further variation would alter the analysis; they did not materially extend the developing patterns, and data collection concluded after Interview 19. The stopping decision concerned analytic sufficiency for the present dataset and research question rather than a claim of exhaustive saturation across the diversity of special education settings in China.

### 2.3. Data Analysis

We analyzed the Chinese-language data using qualitative content analysis, following the preparation, organization, and reporting phases described by Elo and Kyngäs (2008), with inductive open coding and iterative abstraction. This approach supported systematic abstraction of patterned meanings across interviews while allowing analytic categories and relationships to be developed through open coding rather than mapped directly onto the interview-guide domains. Material was coded according to its meaning wherever it appeared in an interview, allowing an account elicited in one area to contribute to another when participants linked the issues themselves. Although the interview guide did not directly ask about well-being or sustained engagement, questions about job satisfaction, emotional experiences, career development, and support elicited relevant accounts. During inductive analysis, we identified recurring connections among these accounts; the resulting themes were not specified in advance and represent participants’ perceived experiences and conditions, rather than measured well-being or observed long-term retention.

During preparation, we transcribed all audio-recorded interviews verbatim in Chinese, and two researchers independently checked every transcript against the corresponding recording. Researchers involved in the analysis read the transcripts repeatedly and recorded preliminary observations in analytic memos. Sentences served as the primary meaning units, but surrounding passages were retained whenever necessary to preserve the narrative context, referents, and evaluative stance of participants’ accounts (Graneheim & Lundman, 2004). This procedure was intended to prevent decontextualized coding of isolated sentences.

During organization, J.W. and M.C. independently conducted open coding across the complete transcripts and compared their coding. Differences in code boundaries or interpretation were discussed by J.W., M.C., and L.C. until consensus was reached. Through iterative abstraction, conceptually related codes were grouped and refined into subthemes. M.C. contributed to the development of themes by examining relationships among subthemes across interviews. Related subthemes were then integrated into three themes. S.Z. provided senior methodological oversight during the development of the final analytic structure, and the final themes were agreed upon through discussion among the analysis team. Here, subthemes refer to intermediate groupings of conceptually related coded content, whereas themes refer to broader interpretive patterns developed through further abstraction across subthemes and participants’ accounts. These terms describe successive levels of abstraction within the inductive qualitative content analysis and do not indicate the use of a separate thematic-analysis approach. Analysis moved repeatedly between individual interviews and the full dataset: the team compared how developing subthemes were expressed by different participants, examined differences in emphasis or conditions, and revisited accounts that did not fit an emerging interpretation. Subthemes were revised when cases complicated a proposed pattern or when distinctions among them were not sufficiently clear. In the final analytic stage, relationships among subthemes were compared across participants and across the full dataset. Related subthemes were then integrated into three themes that captured broader patterns connecting career entry, professional capacity and role, and the conditions participants associated with navigating meaning, demands, and support in special education work.

During reporting, researchers involved in the analysis reviewed the final analytic structure and the excerpts supporting each subtheme and theme. Quotations were selected to make recurrent patterns visible and, where the data supported it, to illustrate meaningful variation in how participants understood the same issue. Coding, abstraction, and theme development were completed using the Chinese-language transcripts. Only quotations selected for publication were translated into English after the analytic structure had been established. J.W. translated the selected quotations into English, and M.C. and S.Z. reviewed them against the Chinese originals to preserve their semantic fidelity and spoken character without polishing them beyond what was needed for clarity. Microsoft Excel 2016 was used to organize transcripts, codes, analytic memos, code definitions, participant identifiers, and successive versions of subthemes and themes. An illustrative example of the progression from a meaning unit to an initial code, subtheme, and final theme is provided in Table 3.

### 2.4. Trustworthiness

We supported trustworthiness through independent transcript checking, independent open coding by two researchers, consensus discussions, reflexive memoing, documentation of coding decisions and successive subtheme and theme versions, and repeated comparison of developing subthemes and themes with the complete dataset. The researchers involved in open coding included PhD students in applied linguistics and an associate professor, all of whom had research experience in special education. Particular attention was given to accounts that complicated or qualified emerging interpretations rather than only to excerpts that confirmed them. Contextual information about participants and the study setting is provided to support judgments about transferability. Reporting was guided by the Consolidated Criteria for Reporting Qualitative Research (COREQ; Tong et al., 2007).

The researchers involved in data analysis included PhD students in applied linguistics and an associate professor, all of whom had research experience in special education. These disciplinary backgrounds and research experiences may have shaped the researchers’ assumptions, attention to participants’ accounts, and interpretations. Some participants had prior professional or personal connections with members of the research team. The interviewers held no supervisory or evaluative authority over participants. Reflexive notes and team discussions were used throughout data collection and analysis to examine how the researchers’ backgrounds, prior connections, recruitment routes, and assumptions might have influenced rapport, questioning, disclosure, and interpretation.

### 2.5. Ethical Considerations

The study was conducted in accordance with the Declaration of Helsinki and received institutional ethics approval from the Ethics Review Committee of the School of Foreign Languages and Literature, Shandong University (Approval No. ECSFLLSDU2024-15) before data collection. Before data collection, participants received information about the study and provided written informed consent for participation, audio recording, and publication of anonymized quotations. Participant, school, and location identifiers were removed from transcripts and reports. Audio files, transcripts, and analytic materials were stored in password-protected digital files accessible only to the research team.

## 3. Results

**Figure 1:**
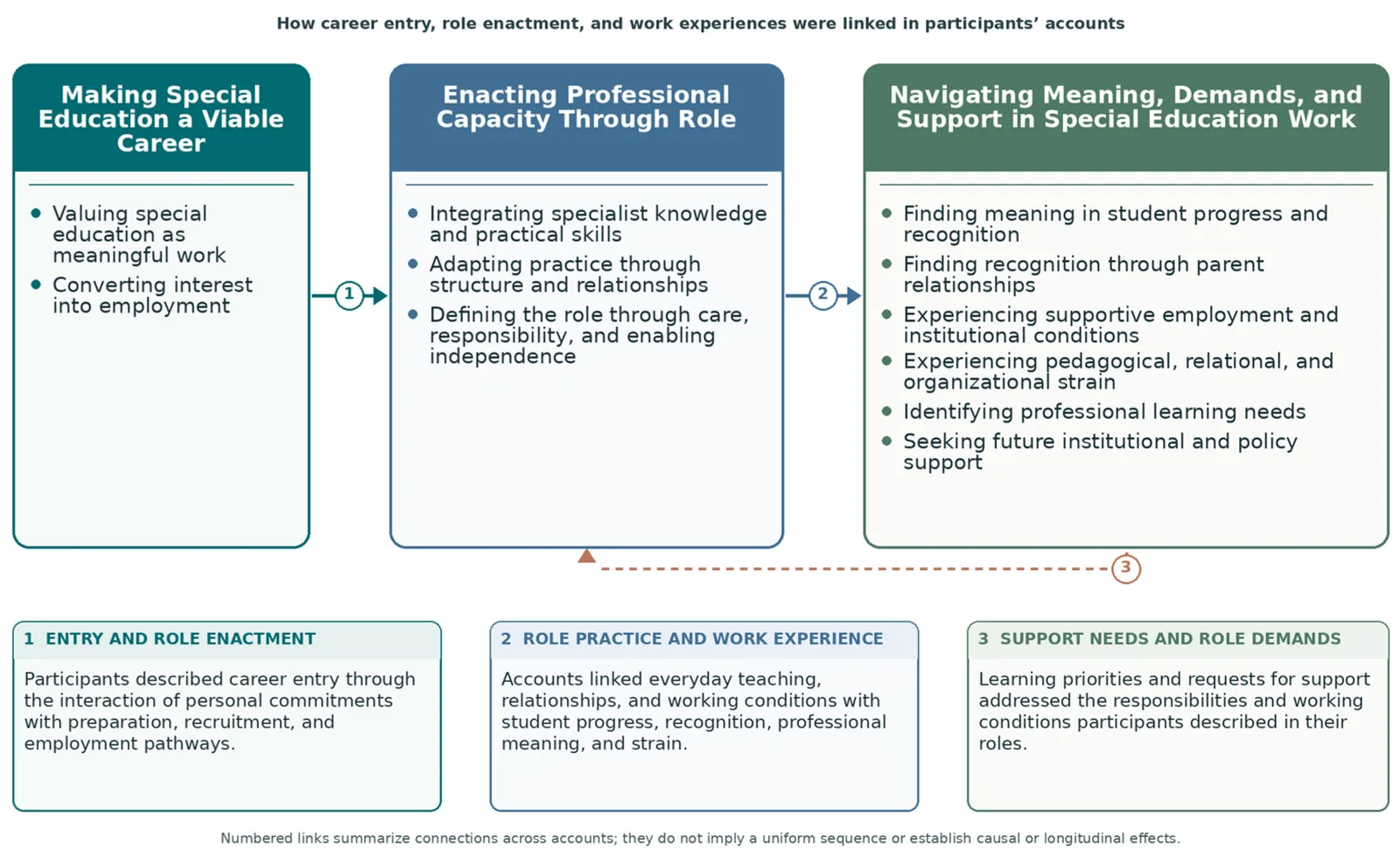
Inter-relationships Among themes in special education teachers’ career accounts. Note: Numbered arrows show how career pathways connect with role enactment, how role practice relates to meaning and strain, and how learning and support needs refer back to role demands. These links summarize participants’ accounts rather than a uniform temporal sequence or causal model.

The analysis generated three themes: making special education a viable career; enacting professional capacity through role; and navigating meaning, demands, and support in special education work. The themes were developed through cross-participant comparison of the coded dataset rather than by reproducing the interview topics one by one. In reporting them, we distinguish patterns identified across multiple accounts from experiences tied to particular participants. We also considered the accounts in relation to years of teaching experience but did not identify consistent differences that warranted distinguishing the findings by career stage. Selected quotations are illustrative rather than a count of how many participants reported each experience. Participant identifiers P1–P19 are used to protect confidentiality. Figure 1 presents the three themes and their subthemes and maps the links among them described in participants’ accounts.

### 3.1. Making Special Education a Viable Career

Entry into special education was described less as a single decision than as an alignment between reasons for valuing the work and opportunities for making it a viable occupation. Participants differed in whether they foregrounded moral responsibility, a personally meaningful encounter, fit with their university preparation, employment stability, or recruitment arrangements. Across these differences, value and opportunity operated as connected rather than mutually exclusive dimensions of career entry.

#### 3.1.1. Valuing Special Education as Meaningful Work

Two accounts illustrate distinct value-based routes into the profession: P13 framed special education as work requiring particular dedication and compassion, whereas P4 traced her interest to an earlier personal encounter with disability.

I think that in China, teaching is generally seen as a very noble profession, and special education is often seen as requiring even more dedication and compassion.

(P13)

P4 instead linked the field to an earlier personal encounter with disability:

I had a classmate in sixth grade who had Down syndrome. That was when I first got the idea to learn more about special education and become a special education teacher. … Later … there happened to be this major, so I applied.

(P4)

These accounts show commitment constructed either through identification with socially valued work or through continuity between earlier experience and later career choice.

#### 3.1.2. Converting Interest into Employment

The accounts of career entry also showed how interest could become employment when prior preparation met concrete opportunity structures. Examples included alignment between academic preparation and available positions, the security associated with public-school employment, and targeted recruitment arrangements. These considerations were not presented simply as ‘extrinsic’ alternatives to commitment; they formed part of how participants retrospectively explained whether an interest in special education became an actionable career pathway under particular labor-market conditions.

When I was about to graduate from college, I saw a high-level talent recruitment policy on the local education bureau website and decided to apply. … A school … needed graduates with my major. I met the requirements, took the examination, and was admitted.

(P1)

P1 shows how prior preparation became occupational entry when recruitment, post availability, and eligibility aligned; P4 similarly linked prior interest to the later availability of special education as a university major. Read with P13, these accounts show value and opportunity as connected rather than competing explanations: what made special education worth doing was linked to pathways that made entry feasible.

### 3.2. Enacting Professional Capacity Through Role

Participants described professional capacity not as a single body of specialist knowledge but as something enacted through judgments about students, relationships, and role purposes. Accounts combined technical knowledge, disability-specific communication systems, repeated instruction, rehabilitation-oriented practice, classroom management, and relational responsiveness. The emphasis varied across teaching contexts, but professional capacity was evaluated by whether teachers could make these resources workable for particular students and toward goals they considered central to the special educator role.

#### 3.2.1. Integrating Specialist Knowledge and Practical Skills

P1 and P17 both emphasized specialist expertise, but they located it at different levels of practice. P1 described professional knowledge in terms of a broad responsibility to identify and respond to students’ support needs, whereas P17 named concrete communication systems required for particular sensory disabilities.

Using what we have learned and our professional skills to help meet their needs and bring out their potential is what we are supposed to do.

(P1)

P17 made this requirement task-specific through disability-specific communication:

For deaf or hard-of-hearing students, you need to know sign language; for blind students, you need to know Braille.

(P17)

The accounts locate specialist capacity across both professional judgment and disability-specific communication, each becoming meaningful in use.

Being patient means teaching them the same things over and over again, day after day, and helping them learn basic daily living skills.

(P13)

P13’s account highlights the repeated nature of this work: patience involved returning to the same daily living skills when progress was slow. In the role accounts below, patience also carried a normative meaning as part of how participants believed a special education teacher should work.

#### 3.2.2. Adapting Practice Through Structure and Relationships

A second strand of professional capacity concerned adapting practice through classroom structure and relationships. P5 emphasized consistent expectations and encouragement as ways of making classroom interaction and teaching more manageable, whereas P15 emphasized care, trust, and attention to students who might not readily express their needs. These accounts shift the analytic focus from possession of techniques toward judgment about how structure and relationships can make teaching and support more workable.

Once I set clear classroom rules and kept them consistent, and gave the students more positive encouragement, things got much better, and teaching became a lot easier.

(P5)

P15 emphasized relational adaptation:

You need to show them greater care … You should also build trust with them. … Therefore, you must carefully attend to their inner needs.

(P15)

These accounts show adaptation as the practical coordination of structure and relationships rather than the application of isolated techniques.

#### 3.2.3. Defining the Role Through Care, Responsibility, and Enabling Independence

Participants also defined professional capacity through a normative understanding of role. Whereas the preceding accounts emphasized what effective practice required, these accounts addressed the kind of professional teachers believed they should be and the purposes their work should serve. Two linked orientations were visible: responsibility, patience, and care as standards for practice, and an enabling orientation toward students’ greater independence.

I would count myself as one of those special education teachers who are highly responsible. … I have been working steadily and diligently, without expecting anything in return.

(P3)

I really enjoy spending time and playing with the children. … I guess you could say I am a teacher with quite a bit of patience and compassion.

(P2)

P3 emphasized responsibility, diligence, and endurance; P2 emphasized closeness, patience, and compassion. In both accounts, these qualities functioned as standards for how the role should be performed rather than merely as personal traits.

You have to teach them the same things over and over and help them develop their daily living skills.

(P14)

P14 extended this normative orientation to role outcomes: repeated teaching was warranted by its contribution to students’ self-care. Across the three accounts, standards for teacher conduct were linked to an enabling purpose of greater student independence.

### 3.3. Navigating Meaning, Demands, and Support in Special Education Work

The third theme captured how participants described meaning, demands, and support in their special education work. Participants linked meaning to student development and recognition, strain to pedagogical, relational, organizational, and emotional demands, and continued development to learning opportunities and institutional support. These dimensions often appeared together rather than as isolated experiences.

#### 3.3.1. Finding Meaning in Student Progress and Recognition

Student development recurred as a source of professional meaning, although participants used different reference points. P2 and P13 emphasized small or incremental gains, while P5 described students’ gradual progress and later academic attainment. In other specific accounts, accomplishment was associated with students’ public participation (P11) or a former student’s recognition (P7).

It took me ages to teach her this small step. But after daily practice last year, she could finally do it on her own. I am glad that our slow teaching worked.

(P2)

When they perform on stage, and I direct from the audience, I feel a strong sense of accomplishment.

(P11)

Every small change and tiny improvement made by the children lets me realize that what I do is meaningful and worthwhile.

(P13)

Thus, professional accomplishment was recalibrated around educationally consequential change, even when progress was minimal or slow, whether visible in self-care, public performance, or incremental development.

Through their own efforts, the students have learned more and more, and some of them have even gone on to college. You can really see them making progress little by little.

(P5)

On Teachers’ Day, a former student sent me a letter thanking me for staying with him in the hospital years ago.

(P7)

P5 and P7 extend this meaning beyond immediate classroom episodes: later progression and former-student recognition made earlier effort retrospectively consequential.

#### 3.3.2. Finding Recognition Through Parent Relationships

The accounts below illustrate two ways parent relationships could be associated with professional meaning. P4 described parents’ praise as explicit confirmation of professional value, whereas P10 linked parental understanding and satisfaction to the work of resolving conflict and communicating patiently. Recognition could therefore appear as direct affirmation, while positive parent responses could also be linked to successful relationship management.

What makes me most proud in my work is the consistent praise I have received from many parents. Their sincere recognition always encourages me a lot.

(P4)

I try to deal with these conflicts as well as I can and talk things through with the parents patiently. If I can ease some of their worries and handle the problems properly, they are usually more understanding and more satisfied.

(P10)

P10 shows that recognition need not be treated merely as an external reward; in this account, parental understanding and satisfaction were produced through relational work. This also helps explain why family communication could affirm professional capacity in some accounts yet become a source of strain in others.

#### 3.3.3. Experiencing Supportive Employment and Institutional Conditions

The two accounts presented here describe positive or qualified evaluations of employment and institutional conditions. P13 evaluated compensation comparatively and conditionally rather than as fully satisfactory, while P3 emphasized professional-development opportunities and favorable local policy. These accounts show how practical and institutional supports could form part of a positive career experience without implying that current conditions were fully satisfactory.

Given my current abilities, I’m fairly satisfied with what I’m paid. Of course, if possible, I still hope special education teachers can receive better support and benefits in the future.

(P13)

The school gives us good opportunities for professional development, and the local government also has some supportive policies for us.

(P3)

Positive evaluation was therefore qualified rather than unconditional: support could be experienced as adequate in one domain while participants still identified room for improvement.

#### 3.3.4. Experiencing Pedagogical, Relational, and Organizational Strain

Strain appeared across several domains: difficulty implementing intended teaching practices, slow student progress, challenging parent issues, non-instructional administrative work, and the emotional effort required to persist. Four participants differed in where they located the difficulty and in the work they believed was necessary to manage it.

A lot of teaching measures are still just talked about, but they are not really carried out well in practice.

(P3)

It can be really overwhelming to deal with students who repeatedly make little progress or with difficult issues involving parents. … I treat teaching as my job, do my duty well, and comfort myself that I have tried my best.

(P6)

Much of my energy is occupied by trivial administrative work. … I am often assigned extra tasks … that have little to do with teaching.

(P10)

Daily work often leaves me feeling stressed and down, so I tell myself to slow down and take a break. … I know I need to keep going.

(P12)

These accounts show that self-regulation occurred within rather than outside the work conditions generating strain. Workload and implementation concerns reappeared in participants’ future development and support needs, linking present strain to the next two subthemes.

#### 3.3.5. Extending Practice Through Professional Learning

Development priorities varied with participants’ responsibilities and perceived gaps in current practice. The three accounts presented here show different priorities: P4 emphasized play-based teaching and language rehabilitation, P5 focused on individualized planning, and P11 highlighted AI-assisted tools while also describing a role in enabling colleagues to explore them. The differences show that professional-development priorities were role- and context-sensitive rather than uniform across participants.

I hope to improve my skills in play-based teaching and language rehabilitation … so that students can enjoy learning, learn better, and become happier.

(P4)

I want to understand each child better, know what they really need, and then make an individualized education plan for them.

(P5)

It is important to encourage teachers to explore and use AI-assisted tools. … I can guide some of the teachers in the school to explore this area actively.

(P11)

Thus, extending practice involved different forms of learning depending on what participants were trying to accomplish in their current roles.

#### 3.3.6. Seeking Future Institutional and Policy Support

Future support needs operated across multiple institutional and policy levels. The four accounts presented here identify distinct institutional and policy needs: P7 described geographic constraints on access to higher-level resources, P6 emphasized salary administration and protection of teaching time, P14 called for coordinated regional development, and P13 questioned appraisal criteria that did not appear sufficiently tailored to special education work. These accounts located future professional work and development within a wider infrastructure of resources, workload, evaluation, and policy rather than solely within teacher motivation.

Because we are in a small county, even though we want to do this, we often do not have access to higher-level resources.

(P7)

P7 shows that motivation to improve practice could be constrained by geography and resource distribution.

Do not delay salary payments—they should be paid on time. … I hope my work can remain mainly student-centered, without having to handle all the miscellaneous tasks that are passed down.

(P6)

I hope different regions can have a coordinated development plan … including improvements in teachers’ salaries.

(P14)

P6 and P14 located needed support at different levels: daily work organization and timely remuneration versus regional coordination. Both positioned material and policy arrangements as part of the infrastructure surrounding professional work.

Could the authorities separate special education teachers from general education teachers and establish an evaluation system tailored specifically to us?

(P13)

P13 added appraisal as a further institutional concern, suggesting that general evaluation criteria were perceived as insufficiently tailored to the distinctiveness of special education work.

The connections among career domains varied across participants. P4 traced her interest in special education to a classmate with Down syndrome and later applied when a special education major became available. In discussing her current development needs, she identified play-based teaching and language rehabilitation as priorities for helping students engage and learn. P5 described using consistent classroom rules and encouragement to make instruction more manageable, viewed students’ subsequent progress as meaningful, and expressed a desire to strengthen her ability to develop individualized education plans. P13’s account brought together patient, repeated instruction in daily-living skills, the significance she attached to small gains in students’ development, and her concerns about compensation and appraisal. P6 described the strain of slow student progress and parent-related difficulties and also called for more student-centred work and fewer unrelated duties. Therefore, these accounts show how career entry, role enactment, meaning, professional learning, strain, and support appeared in different combinations within individual teachers’ career narratives; no single account represented every domain or a uniform career sequence.

## 4. Discussion

This study shows how participants linked commitments, roles, rewards and demands, learning, and institutional conditions within career accounts. The findings suggest that these topics can be considered in relation to one another when interpreting how practicing SETs described their work, but they do not establish a common career sequence or demonstrate effects on retention. Participants’ accounts linked viable entry pathways and role-enacted professional capacity with relational and institutional conditions they associated with making work meaningful and manageable. For settings comparable to those represented in this sample, these connections may point to the value of considering recruitment, preparation, professional learning, and workforce support together.

The entry findings complicate a simple contrast between value-based and pragmatic motives. Prior work identifies social contribution, intrinsic value, cultural influences, self-perceptions, professional growth, and career opportunity as reasons for entering or developing within special education (Almughyiri, 2025; Lindqvist et al., 2021; Valdenegro-Fuentes & González-Sanzana, 2024). This study adds a practicing-teacher perspective: participants retrospectively linked commitment and personally salient experiences with prior preparation, stable employment, and targeted recruitment, showing how interest became a feasible occupation through concrete opportunity structures. Participants linked personal commitment and salient experiences with preparation, stable employment, and recruitment opportunities, suggesting that entry reflected both motivation and available pathways. Longer-serving teachers described secondary normal-school preparation or designated special-education opportunities, whereas more recent entrants referred to university preparation and advertised posts; this sample-specific contrast does not establish a cohort-wide shift. These accounts complement research on preservice special educators’ identity negotiation (Lin, 2026) by illustrating how practicing teachers linked recruitment and preparation.

The findings also suggest that skill inventories alone may not fully capture how participants understood special educator capacity. Preparation research emphasizes complex knowledge, instructional repertoire, collaboration, interaction, and alignment between preparation and classroom practice (Brownell et al., 2010; Takala et al., 2023; Theobald et al., 2022). However, participants judged specialist, pedagogical, and relational capacities by what they enabled teachers to accomplish for students, linking professional capacity to role purposes such as access, development, and independence. This role orientation also offers one way to interpret the relationship between capacity and professional identity. Special educators’ identities can shift with service conditions, values, agency, and expectations (Ellis-Robinson et al., 2025; Lin, 2026). Here, responsibility and care functioned as standards for role performance, while participants defined the role partly through what it should enable students to do. Thus, participants’ accounts connected professional capacity to expectations about responsibility and student independence, as well as to specific skills.

Participants described sources of professional meaning alongside work-related pressures. Student development and recognition were sources of professional meaning, whereas slow progress, difficult family communication, administrative demands, and uneven resources were described as pressures. These accounts may be relevant to teacher well-being, but they are not direct assessments of well-being, job satisfaction, or burnout. These accounts can be considered alongside prior research on autonomy and job satisfaction and on workload, support, burnout, and attrition (Billingsley & Bettini, 2019; Elsayed et al., 2025; Hester et al., 2020; Olsen & Mason, 2023). Emotional regulation likewise appeared to be embedded in professional work rather than as a stand-alone resilience resource. This interpretation is consistent with evidence that special educators use emotional labor in collaboration with adults (Stark et al., 2026) and with research linking blurred care roles and extra-administrative workload to exhaustion and role strain (Channing & Wilson, 2026; Shen et al., 2025). These accounts suggest that teachers’ reported work experiences should be interpreted in relation to pedagogical, relational, and organizational demands, alongside individual coping and commitment. Moreover, professional learning also linked individual capacity with institutional conditions in participants’ accounts. Gaps in ongoing, collaborative, and contextualized development can affect occupational socialization and identity (Woulfin & Jones, 2021), while Chinese evidence links professional identity, self-efficacy, social support, and professional development (Pi et al., 2024). Participants similarly described learning opportunities as extending practice but shaped by geography, workload, remuneration, and appraisal, making development partly dependent on the conditions under which it can be pursued and applied. Overall, the findings offer a qualitative synthesis of connections participants described among career entry, role-enacted professional capacity, professional learning, and relational and institutional conditions associated with meaningful work. These accounts point to questions for future research about how such connections may vary across teachers and settings, and whether they relate to well-being or retention over time.

### Implications for Teacher Education and Workforce Support

Because this study drew on a convenience sample of 19 practicing SETs from public special education schools, the following implications are provisional and may be most relevant to comparable settings; their applicability to other regions and school types requires further examination. First, participants’ accounts suggest that recruitment and preparation may warrant consideration as connected parts of entry into employment. Preparation programs and employing authorities could align specialist preparation with available roles and stable routes into work, while targeted recruitment may connect eligible candidates with posts. Evidence from Hawaii suggests that financial incentives may reduce special education teacher vacancies in that setting (Theobald et al., 2026). Given differences in recruitment and employment contexts, this finding is cited as a point of comparison, not as evidence that the same approach would have similar effects in China. Continued professional growth may also warrant attention alongside these entry pathways. Second, preservice and in-service learning could integrate specialist, pedagogical, rehabilitation-oriented, and relational capacities rather than treating them separately. This includes individualized planning, disability-specific and family communication, emotional and relational support, and technology-supported practice. Because development priorities varied by role and access to expertise, professional learning could be responsive to teaching responsibilities, student needs, and school context rather than organized as a uniform package. Third, participants’ accounts suggest that organizational conditions may warrant consideration. Schools could protect instructional time, reduce unrelated administrative work, strengthen access to specialist consultation and peer learning, and enable teachers to apply professional learning. Education authorities could also consider compensation, regional access to development, and appraisal criteria sensitive to the distinctive responsibilities and outcomes of special education work. These priorities align with research on working conditions and leadership support (Billingsley et al., 2020) and institutional constraints in Chinese inclusive education (Diao et al., 2024). This perspective may help frame workforce support around work organization as well as individual commitment.

Several limitations should guide interpretation of the findings. First, the sample comprised 19 practicing SETs from 19 public special education schools, with most participants located in northern and eastern China. The study did not include teachers in private schools, inclusive settings within mainstream schools, or former SETs. Because all participants were currently employed, the sample may also reflect survivor or retention bias and may not capture perspectives associated with leaving the profession. Only two participants were men, precluding meaningful gender comparisons. In addition, because only one teacher was recruited from each school and no independent school-level data were collected, institutional conditions reflect participants’ perceptions rather than verified differences among schools. The findings should therefore be understood as analytically developed accounts from a bounded sample rather than as representative of the full diversity of special education contexts in China. Second, the study relied on retrospective self-reported interviews. Participants’ accounts of career-entry decisions, particularly those made many years earlier, may be affected by recall bias. Because the interviews also addressed professional responsibility, compassion, effectiveness, and commitment, some responses may have been influenced by social desirability. These accounts provide insight into how teachers interpret their career experiences but cannot establish how reported practices unfolded in classrooms or how school leaders, colleagues, students, or families understood the same circumstances. Because all interviews were conducted online via Tencent Meeting, the findings may reflect features of online interviewing and may not capture interactional dynamics that could emerge in face-to-face interviews. Future research could triangulate teachers’ accounts with observations, documents, and perspectives from other stakeholders. Finally, the cross-sectional design captures career sense-making at one point in time and does not show how professional capacity, experiences of meaning and strain, or support needs change across career stages. Accordingly, the study does not establish changes in work engagement or provide evidence of actual retention over time. Longitudinal studies with more diverse samples could examine how these processes develop across career stages and institutional contexts.

## 5. Conclusions

This study offers a qualitative account of how participating SETs connected career entry; role enactment; and the meaning, demands, and support associated with their work. These connections varied across accounts rather than following a shared career sequence. The findings suggest considerations for teacher preparation and workforce support in comparable settings. Well-being, work engagement, and retention were not directly assessed and warrant further study.

## Figures and Tables

**Table 1:** Participant Characteristics.

ID	Gender	Highest Qualification	Teaching Experience (Years)	Teaching Responsibilities	Field of Study
P1	Female	Bachelor’s degree	5	Mathematics, life skills, and handicrafts	Special education
P2	Female	Bachelor’s degree	27	Group rehabilitation (autism and intellectual disabilities)	Special education (deaf education)
P3	Male	Bachelor’s degree	33	Music	Special education
P4	Female	Bachelor’s degree	3	Life-oriented Chinese	Special education
P5	Female	Secondary normal school diploma	30	Life-oriented Chinese	Special education (deaf education)
P6	Female	Bachelor’s degree	5	Life-oriented Chinese	Special education
P7	Female	Bachelor’s degree	2	Life-oriented Chinese	Special education (pedagogy)
P8	Female	Bachelor’s degree	6	Life-oriented Chinese	Special education
P9	Female	Master’s degree	3	Life-oriented Mathematics	Special education
P10	Male	Bachelor’s degree	3	Physical education, health science	Special education
P11	Female	Master’s degree	2	Life-oriented Mathematics, Chinese language, and art	Pedagogy (special education)
P12	Female	Master’s degree	9	Life-oriented Chinese, history, and sign language	Literature and art studies
P13	Female	Bachelor’s degree	17	Daily life reading	Special education
P14	Female	Master’s degree	4	Upper-secondary Chinese	Chinese language and literature
P15	Female	Master’s degree	3	Life-oriented Chinese	Special education
P16	Female	Bachelor’s degree	9	Folk arts and crafts design	Design
P17	Female	Bachelor’s degree	4	Health care	Rehabilitation
P18	Female	Bachelor’s degree	3	Life-oriented mathematics and health care	Preschool education
P19	Female	Bachelor’s degree	5	Life-oriented Chinese	Special education

Note. “Life-oriented Chinese” and “Life-oriented mathematics” refer to functional language and mathematics curricula used in Chinese schools serving students with intellectual disabilities, emphasizing communication, literacy, numeracy, and application to everyday life. “Secondary normal school diploma” refers to a teacher-training qualification awarded by a secondary-level normal school.

**Table 2:** Semi-Structured interview guide.

No.	Interview Question
1	How do you understand the role and professional identity of special education teachers?
2	What motivated you to become a special education teacher, and how has your teaching career developed?
3	What qualities make an effective special education teacher, and how do you perceive your own professional practice and job satisfaction?
4	What are your professional goals, development needs, and expectations for external support?
5	Could you describe your instructional practices, classroom interactions, and emotional experiences in teaching?

Note. The interview questions are presented here as English translations; all interviews were conducted in Chinese.

**Table 3:** Illustrative progression from meaning unit to final theme.

Interview Meaning Unit	Condensed Meaning	Initial Code	Subtheme	Final Theme
“I had a classmate in sixth grade who had Down syndrome. That was when I first got the idea to learn more about special education and become a special education teacher.” (P4)	An early personal encounter with a classmate with Down syndrome prompted P4’s interest in special education and the possibility of becoming a teacher.	Personal encounter with disability sparked vocational interest in special education.	Valuing Special Education as Meaningful Work	Making Special Education a Viable Career

*Note. This example illustrates one analytic pathway. The subtheme and final theme were developed by comparing accounts across participants; they were not derived from this excerpt alone.*

## Data Availability

The interview data are not publicly available because of participant confidentiality and the conditions of ethical approval.
